# The presence of circulating genetically abnormal cells in blood predicts risk of lung cancer in individuals with indeterminate pulmonary nodules

**DOI:** 10.1186/s12890-023-02433-4

**Published:** 2023-06-05

**Authors:** Shahram Tahvilian, Joshua D. Kuban, David F. Yankelevitz, Daniel Leventon, Claudia I. Henschke, Jeffrey Zhu, Lara Baden, Rowena Yip, Fred R. Hirsch, Rebecca Reed, Ashley Brown, Allison Muldoon, Michael Trejo, Benjamin A. Katchman, Michael J. Donovan, Paul C. Pagano

**Affiliations:** 1 LungLife AI, Inc, 2545 W. Hillcrest Drive, Suite 140, Thousand Oaks, CA USA; 2 Department of Interventional Radiology, The University of Texas MD Anderson Cancer Center, Houston, TX USA; 3 Department of Radiology, Icahn School of Medicine at Mount Sinai, New York, NY USA; 4 Icahn School of Medicine, Center for Thoracic Oncology, Tisch Cancer Institute at Mount Sinai, New York, NY USA; 5 Department of Pathology, Cancer Institute, Icahn School of Medicine at Mount Sinai, New York, NY USA

**Keywords:** Pulmonary nodules, Indeterminate nodules, Liquid biopsy, Lung cancer, Early detection

## Abstract

**Purpose:**

Computed tomography is the standard method by which pulmonary nodules are detected. Greater than 40% of pulmonary biopsies are not lung cancer and therefore not necessary, suggesting that improved diagnostic tools are needed. The LungLB™ blood test was developed to aid the clinical assessment of indeterminate nodules suspicious for lung cancer. LungLB™ identifies circulating genetically abnormal cells (CGACs) that are present early in lung cancer pathogenesis.

**Methods:**

LungLB™ is a 4-color fluorescence *in-situ* hybridization assay for detecting CGACs from peripheral blood. A prospective correlational study was performed on 151 participants scheduled for a pulmonary nodule biopsy. Mann-Whitney, Fisher’s Exact and Chi-Square tests were used to assess participant demographics and correlation of LungLB™ with biopsy results, and sensitivity and specificity were also evaluated.

**Results:**

Participants from Mount Sinai Hospital (n = 83) and MD Anderson (n = 68), scheduled for a pulmonary biopsy were enrolled to have a LungLB™ test. Additional clinical variables including smoking history, previous cancer, lesion size, and nodule appearance were also collected. LungLB™ achieved 77% sensitivity and 72% specificity with an AUC of 0.78 for predicting lung cancer in the associated needle biopsy. Multivariate analysis found that clinical and radiological factors commonly used in malignancy prediction models did not impact the test performance. High test performance was observed across all participant characteristics, including clinical categories where other tests perform poorly (Mayo Clinic Model, AUC = 0.52).

**Conclusion:**

Early clinical performance of the LungLB™ test supports a role in the discrimination of benign from malignant pulmonary nodules. Extended studies are underway.

**Supplementary Information:**

The online version contains supplementary material available at 10.1186/s12890-023-02433-4.

## Introduction

In 2022, the estimated number of new lung cancer cases in the United States is 236,740 with 130,180 deaths [1]. Identification of lung cancer at earlier stages results in more favorable prognoses and outcomes [2]. Lung cancer mortality has been slowly declining, mostly attributed to improved treatments and earlier diagnosis [1, 3]. Computed tomography (CT) was found to be capable of identifying lung cancers at an earlier, more curable stage [4], which was further supported by The National Lung Screening Trial (NLST) and Nederlands–Leuvens Longkanker Screenings Onderzoek (NELSON) studies that demonstrated a reduction in lung cancer-specific mortality when CT was used for screening in defined high-risk populations [5, 6]. Early-stage lung cancer continues to be challenging to detect due to low adherence to lung cancer screening guidelines and because early-stage disease is typically asymptomatic [1].

The majority of early-stage lung cancers are initially identified as indeterminate pulmonary nodules (IPNs), and an estimated 1.5 million IPNs are identified in the US each year using CT [7]. Guidelines, such as those from the American College of Chest Physicians (ACCP) and Fleischner Society are generally in alignment for low- or high-risk nodules, where the pre-test probability for lung cancer is < 5% or > 65%, respectively [8, 40]. For intermediate risk IPNs (5-65% pre-test probability for lung cancer), guidelines are poorly aligned and these nodules represent the most challenging to evaluate [9]. It is estimated that > 40% of biopsies of CT-identified IPNs are negative for lung cancer [10], unnecessarily exposing individuals to invasive biopsy procedures, where approximately 20% of patients experience adverse events, including infection, pneumothorax, hemorrhage and even death from the procedure [10–12]. This highlights the need for an improved noninvasive method that provides additional information with higher confidence for individuals with IPNs.

Diagnosing cancer using blood has significant advantages: the process is minimally invasive and with very low risk of associated complications [11, 13]. Whole blood is a complex mixture that includes plasma and cell-based components, each of which contains unique biomarkers that can provide complementary information [14]. A main advantage of using blood compared with tissue biopsy is that the specimen is not restricted to a single tumor site, overcoming tumor heterogeneity and allowing more diverse sampling of circulating components from the tumor, such as circulating tumor cells (CTCs), circulating tumor DNA (ctDNA), and immune cells.

Laboratory and clinical investigations have revealed that lung epithelial cells have a reported capacity for motility thought to be derived from evolutionary requirements to repair damaged epithelium, as the need for motility-related wound healing likely preceded motility related to metastasis and carcinogenesis [15–17]. In lung cancer the early appearance of metastatic behavior has been demonstrated [17, 18]. Previous studies demonstrate that CTCs can be identified in patients with stage I lung cancer [18, 19], and those with chronic obstructive pulmonary disease (COPD) at high-risk for lung cancer years before a malignancy is observed radiographically [20]. Early metastatic behavior can be leveraged for early detection of lung cancer using various assays.

Numerous blood-based technologies have emerged to facilitate early detection of lung cancer by identifying RNA, proteins, circulating cell-free DNA (cfDNA), methylated cfDNA, ctDNA, or CTC [21–23]. These technologies are limited by their reliance on broad detection of molecular pathophysiological changes typically associated with high tumor burden in later-stage disease and are less likely to accurately detect early-stage lung cancer [21, 22]. Detection of ctDNA has had a marked impact on treatment stratification for late-stage lung cancer and this approach is being reevaluated for early-stage lung cancer. Low tumor cell burden limits the capacity to detect the smallest stage I lung cancers using existing ctDNA profiling methods [24]. Detecting early-stage lung cancer based on the presence of CTCs has been challenging given that CTCs are generally detected in small numbers in approximately 30% of patients with non-small cell lung cancer (NSCLC) [25–27]. Traditional CTC-based assays depend on the presence of epithelial markers to isolate and/or identify CTCs. Metastatic cancer cells commonly undergo epithelial to mesenchymal transition; identification of these CTCs is challenging using traditional approaches [29]. Due to the low sensitivity levels and suboptimal performance of these emerging technologies, they continue to be further optimized for clinical use [21, 30].

Chromosomal instability, a hallmark of cancer, can result in genomic copy number variations (CNVs) that can be readily detected with well-established technologies in individual cells, namely fluorescence in situ hybridization (FISH) [28]. FISH is employed by the LungLB™ test to detect CNVs in circulating genetically abnormal cells (CGACs) enriched from the peripheral blood of individuals with IPNs [28]. Studies have reported the presence of CGACs in individuals with various cancers, including lung cancer, and some of these CGACs have been identified as CTCs [28, 31, 32]. Individuals with cancer, including lung cancer, have been reported to have circulating lymphocytes with cytogenetic abnormalities that are identical to those found in cancerous cells from the primary tumor [28, 33–36]. As FISH is generally a highly specific assay for detecting chromosomal instability that is frequently observed in cancer, this method allows more comprehensive detection of multiple types of CGACs that have been reported to be associated with lung cancer [28, 37].

Here we report the results of a study to assess clinical performance of the LungLB™ test, a liquid biopsy assay that utilizes immunomagnetic depletion combined with FISH, circumventing current cell marker restrictions of traditional CTC-based assays, to detect CGACs in individuals with IPNs. In this study, we evaluate concordance of lung nodule biopsy outcomes with the LungLB™ test results for participants with IPNs identified incidentally or through a lung cancer screening program.

## Materials and methods

### Participant enrollment

All participants meeting eligibility criteria and without preselection were enrolled from MD Anderson Cancer Center in Houston, Texas and the Mount Sinai Health System in New York, New York. Laboratory and statistical personnel were blinded to the results of the biopsy and clinical information. The results of the test did not direct or influence participant care. All sites had institutional review board approval, and informed written consent was obtained from all eligible participants.

Eligible participants were older than 18 years of age and scheduled for percutaneous needle biopsy. There were no restrictions on nodule characteristics. Participants were ineligible if they had a prior (3 year) or concurrent cancer diagnosis of any type, or a lung cancer diagnosis within the past 2 years. Inclusion and exclusion criteria were intentionally kept broad to avoid bias and reflect real-world conditions. This was an all-comers study and all eligible participants were enrolled on a “first-come, first-served” basis. Participants enrolled in this study were followed for 9–24 months following biopsy to confirm malignant or benign diagnosis using standard of care procedures at each site, including CT-based surveillance, repeat biopsy, and/or surgery.

### Peripheral blood Collection

Peripheral blood was collected just prior to the CT-guided needle biopsy procedure. Blood was collected in vacutainer tubes containing blood stabilizer (Streck, Omaha, NE) and shipped overnight to LungLife AI’s Clinical Laboratory Improvement Amendments (CLIA)-certified lab in Thousand Oaks, CA.

### CGAC Enrichment and fluorescence ***in situ*** hybridization

A procedure and training set first describing the 4-probe FISH assay used by LungLB™ has been described previously [32]. Samples received by the CLIA-certified lab were accessioned using 2 unique identifiers. Blood was centrifuged at 1000 x g for 10 min with the brake off. Plasma was transferred to new tubes and stored at -80 ºC. Erythrocytes were removed using an ammonium chloride-based erythrocyte lysis buffer. The remaining leukocytes were quantified using a BD Accuri™ C6 flow cytometer (Becton Dickenson, San Jose, CA) and 5,000,000 leukocytes were transferred to a new tube for magnetic cell depletion. We performed a depletion using the LungLB™ antibody cocktail to remove neutrophils and monocytes.

Ten thousand cells from the cell suspension were transferred to a glass slide using a cytospin instrument. Cells were fixed in Carnoy’s fixative (3:1 solution of methanol and glacial acetic acid) for 30 minutes, followed by treatment with protease (pepsin pH 2, Abbot Molecular, Abbott Park, IL). Four colored FISH probes targeting chromosome locations 3q29 (Green), 3p22.1 (Red), 10p22.3 (Gold) and 10cen (Aqua), which localize to regions previously found to be altered in lung tumors [38], were then added to the microscope slide and a coverslip was affixed using rubber cement. DNA was denatured at 80 ºC for 2 minutes, followed by overnight hybridization at 37 ºC in a humidified chamber for 18 hours. Slides were washed to remove background in 72 ºC wash buffer 1 (0.4x saline-sodium citrate (SSC) buffer/0.3% IGEPAL® CA-360, pH 7.0) for 1 minute followed by room temperature wash buffer 2 (2x SSC buffer/0.1% IGEPAL® CA-360, pH 7.0) for 1 minute. A new coverslip was applied with mounting medium containing 4’,6-diamidino-2-phenylindole (DAPI; Vector Labs, Burlingame, CA) to visualize cell nuclei.

### Image Acquisition and Analysis

Slides containing cells were imaged using a Bioview Allegro-Plus microscope system (Bioview USA, Billerica, MA). Images were acquired using a 60x objective (1.35 NA oil immersion on UPlanSapo, Olympus, Bartlett, TN) and a FLIR Grasshopper 3 monochrome camera (12-bit, 2448 × 2048 pixels, 3.4 μm pixel size, Edmund, Barrington, NJ) controlled using Bioview Duet software. All cells were imaged with 21 transverse sections spanning 0.65 μm.

Objects were classified by the Bioview Duet software according to probe copy number variation. Normal cells have 2 spots in all 4 color channels (Red, Green, Aqua, and Gold) and CGACs have a gain of spots in ≥ 2 color channels. Advanced CGACs were CGACs with the following specific anomalies: 4 spots in 2 color channels (4 × 2 Advanced CGAC), or a gain of spots in 2 color channels plus any loss of spots in 2 color channels (Double Deletion Advanced CGAC). Though Advanced CGACs can be considered a subtype of CGACs, classification of cells as a CGAC or Advanced CGAC was mutually exclusive in this study. Cells binned in the CGAC classes by the BioView Duet software were analyzed by a licensed technician who verified each cell. CGAC counts were normalized by dividing the CGAC count by the total number of cells analyzed and multiplying by 10,000. A minimum of 10,000 cells were analyzed per participant. Total CGAC count, total cell count, and normalized CGAC counts were sent for unblinding for each participant.

### Statistics

Statistical significance of clinical factor data was determined using the Mann–Whitney test (two-tailed, 95% confidence interval), Fisher’s Exact test (two-tailed, 95% confidence interval), and Chi-Square test (two-tailed, 95% confidence interval). To determine the significance of different CGAC subtypes between participants with malignant and benign diagnosis, the Fisher’s Exact Test (two-tailed, 95% confidence interval) or Chi-Square test (two-tailed, 95% confidence interval) were used depending on the sample size. A *P* < .05 was considered statistically significant in all analyses.

The nuclear area of cells was measured from DAPI stained cells using the Bioview software and data was exported to Prism (GraphPad Prism 9.3.0, San Diego, CA) for analysis of descriptive statistics.

To establish sensitivity, specificity, and area under the curve (AUC) for the LungLB™ test, we used the Youden Index to define a threshold of 2.47 CGAC cells per 10,000 cells. Receiver operating characteristic (ROC) analysis was performed in Prism using normalized CGAC counts from participants with malignant and benign nodules. For ROC analyses that were weighted for Advanced CGACs, the normalized CGAC ratio for samples containing Advanced CGACs was automatically set to 10.0 and declared qualitatively positive, regardless of the original CGAC ratio.

For the multivariate analysis, the predictors of interest (CGAC characteristic, age, gender, smoking status, COPD/Emphysema, subsolid/nonsolid nodule, nodule location, nodule size, and cancer history) were entered into a univariate and multivariate logistic regression model with cancer diagnosis as the outcome. The AUCs were compared using DeLong tests (two-sided). Complete case analysis was performed, and all analyses were completed in R v4.1.0 (R Foundation for Statistical Computing, Vienna, Austria).

Additional statistical analysis was performed to determine the calibration of the LungLB™ unweighted and weighted assay. Calibration curves were created by plotting predicted probabilities from the logistic regression model against the dichotomized diagnosis outcome and fitting the curve using the LOESS smoother. Brier score, Spiegelhalter scores and *P* values were calculated using the ‘rms’ package in R.

## Results

Between December 2018 and February 2021, 182 participants were enrolled in the study; 19 participants were excluded from analysis, either due to an indeterminate biopsy result (n = 14) or having a sample that was unable to be processed due to clotting or damage (n = 5) (Figure S1). Samples from 12 of the 163 participants who met the initial eligibility criteria did not pass the assay quality control and these participants were excluded. A total of 151 participants met all study inclusion criteria. Of these 151 participants, 112 participants were diagnosed with malignant lung lesions and 39 participants with benign lung lesions based on blinded nodule biopsy results. Participant and disease characteristics commonly used in malignancy prediction models were compared in participants with benign versus malignant nodules and no statistically significant differences were found (Table 1). We also utilized the Mayo Clinic risk model to predict the probability of malignancy in this study cohort. With the Mayo Clinic Model, the majority of participants (107 [70.9%] of 151 participants) in our study fell into the intermediate-risk category as defined by ACCP guidelines (pre-test probability of malignancy between 5% and 65%), for which current management guidelines are not well-standardized and represents the most challenging diagnostic group.

**Table 1:** Participant Characteristics (Negative for Lung Cancer, atypical cells, plural tumor, bronchiectasis)

Category, n (%)	Overall (N = 151)		*P* value
	**Malignant Nodules** **(n = 112)**	**Benign Nodules** **(n = 39)**	
**Sample n/N, %**	74.2	25.8	N/A
**Gender**			
Male	52 (46.4)	17 (43.6)	0.8525*
Female	60 (53.6)	22 (56.4)	
**Age, Median (Range), y**	70.0 (40.0)	71.0 (45.0)	0.7838^**†**^
**Smoking Status**			
Current	18 (16.1)	6 (15.4)	0.9115^§^
Former	70 (62.5)	26 (66.7)	
Never	23 (20.5)	7 (17.9)	
Unknown	1 (0.9)	0 (0)	
**Median Pack Years, y**	25.0	22.8	0.9083^†^
**Nodule Location**			
Upper Lobe	70 (62.5)	25 (64.1)	0.999
Non-upper lobe	42 (37.5)	14 (35.9)	
**Nodule Size**			
<1 cm	16 (14.3)	13 (33.3)	0.0654^§^
1 cm to <2 cm	41 (36.6)	13 (33.3)	
2 cm to <3 cm	32 (28.6)	7 (18.0)	
≥3 cm	23 (20.5)	6 (15.4)	
**Malignant Lesion Type**			
Adenocarcinoma	73 (65.2)	-	
Squamous Cell Carcinoma	17 (15.2)	-	
Small Cell and Neuroendocrine	17 (15.2)	-	
Other	5 (4.6)	-	
**Cancer Stage**			
I	76 (67.9)	-	
II	16 (14.2)	-	
III	7 (6.3)	-	
IV	8 (7.1)	-	
Unknown	5 (4.5)	-	
**Benign Lesion Type**			
Infectious Etiology	-	8 (20.5)	
Non-infectious Inflammation	-	5 (12.8)	
Hamartoma	-	3 (7.7)	
Scar	-	3 (7.7)	
Decrease in nodule size	-	4 (10.3)	
Other**	-	16 (41.0)	
**Risk Stratification Based on** **Mayo Clinic Model**			0.0883^§^
Low Risk (< 5%)	17 (15.2)	3 (7.7)	
Intermediate Risk (5-65%)	74 (66.1)	33 (84.6)	
High Risk (> 65%)	21 (18.7)	3 (7.7)	

* Fisher’s Exact.

^†^ Mann–Whitney.

^§^ Chi-square test.

**Other includes biopsy results of no malignant cells present, atypical cells, plural tumor, or bronchiectasis, which were followed between 9 and 24 months without diagnosis of malignancy.

### CGAC characterization

**Fig. 1:**
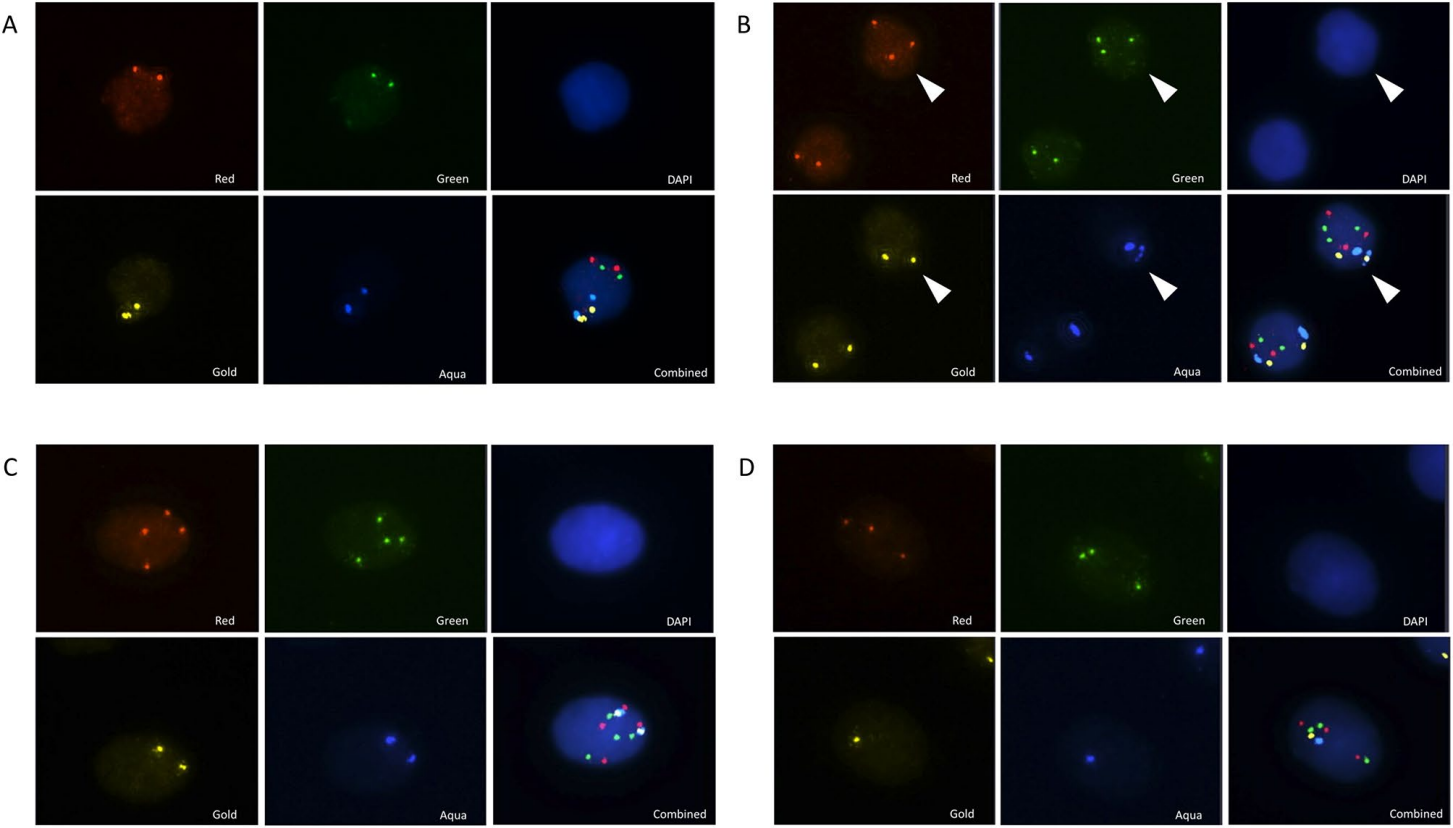
Representative fluorescence microscopy images from the LungLB™ test demonstrating (A) a normal WBC with 2 spots in the Red (3p22.1), Green (3q29), Aqua (10cen), and Gold (10q22.3) color channels, (B) a CGAC, indicated by the white arrow, with an extra spot in the Red and Green channels, (C) an Advanced CGAC with 4 spots each in the Red and Green channels (4 × 2 Advanced CGAC), and (D) an Advanced CGAC with an extra spot in the Red and Green channels and a spot loss in the Gold and Aqua channels (Double Deletion Advanced CGAC). Cell nuclei are visualized in the DAPI channel. Abbreviations: CGAC, circulating genetically abnormal cell; DAPI, 4′,6-diamidino-2-phenylindole; WBC, white blood cell.

The LungLB™ test utilizes a 4-color FISH assay to detect CNVs in cells from peripheral blood and identify CGACs. Figure 1a provides representative images of a normal white blood cell (WBC) with a diploid copy number per FISH probe, indicated by 2 spots detected per probe color channel (Red, Green, Aqua, and Gold). The LungLB™ test identifies CGACs based on a gain of spots in ≥ 2 color channels. Though there existed cells with spot abnormalities in a single-color channel, only cells with a gain in spots in ≥ 2 color channels were classified as CGACs. Examples of representative CGAC that were identified and used to differentiate benign from malignant participants are shown in Fig. 1b, c, and d. Advanced CGACs were detected in 44 (39.3%) samples from participants with confirmed malignant nodules vs. 4 (10.3%) samples from participants with confirmed benign nodules (Table 2). There was also a greater number of CGACs (*P* = .004) and Advanced CGACs (*P* = .001) identified among malignant versus benign participant samples.

**Table 2:** CGAC and Advanced CGAC Cell Counts in Malignant and Benign Participant Samples

n (%)	Total Participant Samples (n = 151)	Malignant Participant Samples (n = 112)	Benign Participant Samples (n = 39)	*P* value (Malignant vs. Benign Participant Samples)
**Advanced CGAC**	48 (31.8)	44 (39.3)	4 (10.3)	0.001*

Abbreviations: CGAC, circulating genetically abnormal cell

^*^ Chi-Square Test

To further characterize CGACs, we performed morphological analysis to compare nuclear area to the average nuclear area of each respective participant’s normal WBCs. CGACs exhibited highly variable nuclear areas (range, 0.547x-2.776x relative to normal WBC), and we found that the mean nuclear area of Advanced CGACs was approximately 21.1% larger than the average WBC (95% CI, 13.6-28.6%) and approximately 13% larger than CGACs (*P* < .0001) (Figure S2 and Table S1).

### LungLB™ Test Performance

**Fig. 2:**
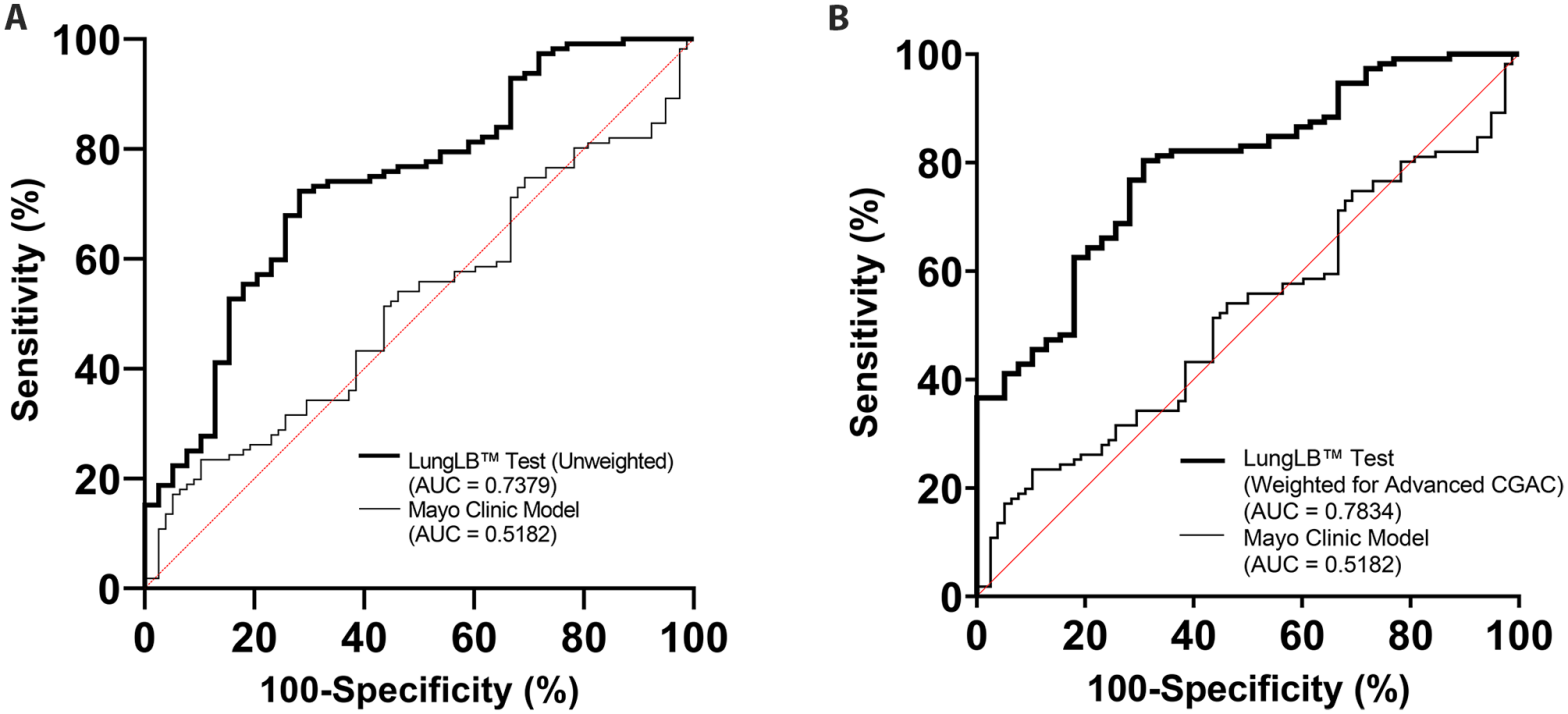
ROC analysis of (A) the LungLB™ test without weighting of Advanced CGACs (AUC = 0.74) and (B) the LungLB™ test with weighting of Advanced CGACs (AUC = 0.78) compared with the Mayo Model (AUC = 0.52). Abbreviations: AUC, area under the curve.

We evaluated the overall performance and diagnostic efficacy of the LungLB™ test by plotting ROC curves in comparison with the ROC from the Mayo Clinic Model and positron emission tomography (PET) scan results. The Mayo Clinic Model was chosen as a comparator because it is the most frequently used probability model and was developed using the general population, rather than an isolated cohort, reflecting the population for which LungLB™ is intended to be offered [39]. ROC analysis of the full data set consisting of 151 participants revealed an AUC of 0.74 (95% CI, 0.66–0.84; *P* < .001) with 67.9% sensitivity and 74.4% specificity, compared with an AUC of 0.52 using the Mayo Clinic Model and an AUC of 0.57 using the PET imaging results available (Fig. 2A; Table 3). We observed that Advanced CGACs were more highly associated with malignant lung cancer diagnosis, and an additional ROC analysis where Advanced CGACs were weighted more heavily revealed an AUC of 0.78 (95% CI, 0.70–0.87; *P* < .0001) with 77% sensitivity and 72% specificity, compared with an AUC of 0.52 using the Mayo Clinic Model and an AUC of 0.57 using the PET imaging results available (Fig. 2B; Table 3). To ensure that our LungLB™ assay is in calibration we plotted a calibration curve using both the unweighted and weighted assays (Figure S3). For each calibration plot, the Brier score was calculated to determine fit, where a score closer to 0 represents a good fit (0.163 [unweighted] and 0.158 [weighted]). Using the Brier score and Spiegelhalter z-score we calculated a Spiegelhalter p-value to test for misclassification. A *P* < .05 would indicate our model is poorly calibrated. LungLB™ assay has a *P* value of 0.88 (unweighted) and 0.81 (weighted) indicating that the LungLB™ assay is calibrated properly.

**Table 3:** LungLB™ Test Performance by Participant Characteristic

Category	n	AUC	Sensitivity (%)	Specificity (%)
All Samples (Mayo Clinic Model)	151	0.5182	23.4	89.7
Samples with Available PET Scan	79	0.5674	67.2	.4
All Samples (Unweighted)	151	0.7379	67.9	74.4
All Samples (Weighted for Advanced CGACs)	151	0.7834	76.8	71.8
** Nodule Size**				
< 2 cm	83	0.8269	83.9	69.5
≥ 2 cm	68	0.7049	69.1	69.2
**Nodule Consistency**				
Solid	92	0.7215	65.2	65.2
Subsolid	41	0.8952	83.3	80.0
**Cancer Stage**				
I	76	0.7945	79.0	74.4
II-IV	31	0.6716	61.3	71.8
Unknown	5	0.7333	80.0	71.8
**Malignant Lesion Type**				
Adenocarcinoma	73	0.7875	72.6	74.4
Squamous Cell Carcinoma	17	0.8250	88.2	71.8
Small Cell and Neuroendocrine	17	0.7541	76.5	71.8
Other	5	0.6970	60.0	69.2
**Smoking History**				
Ever	120	0.7929	76.4	71.0
Never	30	0.8075	78.3	85.7

Abbreviations: AUC, area under the curve, CGAC, circulating genetically abnormal cell, PET, positron emission tomography. Results from all 39 benign nodules were used as the comparator for cancer stage and malignant lesion type

Diagnostic efficacy of the LungLB™ test was also evaluated for various participant characteristics, including nodule size and consistency, lung cancer subtype and stage, and smoking history (Table 3). The LungLB™ test demonstrated robust performance for each participant characteristic category. Superior LungLB™ test performance was observed with nodules < 2 cm in size (AUC = 0.83) vs. ≥ 2 cm in size (AUC = 0.70), sub/nonsolid nodules (AUC = 0.90) vs. solid nodules (AUC = 0.72) and may be better in stage I (AUC = 0.80) vs. stage II-IV disease (AUC = 0.67). Comparable test performance was observed between lung cancer subtypes (adenocarcinoma, AUC = 0.79; squamous cell carcinoma, AUC = 0.83; and small cell and neuroendocrine, AUC = 0.75) and smoking history (Ever, AUC = 0.79; and Never, AUC = 0.81).

To evaluate the independent and associated contribution of readily available clinical factors (e.g., age, sex, smoking history, presence of COPD/emphysema, nodule type, location and size, and cancer history) we performed a stepwise multivariable analysis (Table S2). CGAC were the strongest independent predictor of malignancy in this study. Although there were no statistically significant differences when we compared the AUCs across the different models when combining CGAC with clinical factors, comparison of Model 3 (Clinical Factors) vs. Model 5 (Clinical Factors + Weighted CGAC Ratio) revealed a *P* = .053, which suggests that combining CGAC to clinical factors may improve the diagnostic efficacy of the LungLB™ test compared with when clinical factors are used alone (Table S3).

To describe how LungLB™ may be useful clinically, we describe three example cases in Table 4 and how LungLB™ may have saved critical time in determining a final treatment regimen. In each of the three cases the Mayo Risk Score (pretest probability) fell into or near the intermediate risk category (25%, 67%, and 47%), representing challenging cases to evaluate. All three cases had a negative or indeterminate biopsy result. LungLB™ results indicated an increased risk for malignancy for all three cases. Following the initial biopsy Cases 1 and 2 underwent imaging-based follow-up, followed by surgical treatment and final diagnosis of Stage I Adenocarcinoma and Stage IA3 Adenocarcinoma, respectively. Case 3 underwent a follow-up biopsy in May 2020 on the lymph nodes, in which one tested positive for small cell lung cancer.

**Table 4:** Example Case Studies

Case	Mayo Risk Score	Initial CT/ Nodule Size	Biopsy Date/ Result	LungLB™ Date/ Result	Surgery Date/ Result	Days LungLB™ may have saved
**1**	25%	Nov 2018/ 1.3 cm	Jan 2020/ Negative	Jan 2020/ 6.97	Jan 2021/ Stage 1 Adenocarcinoma	365
**2**	67%	July 2019/ 2.6 cm	Aug 2019/ Indeterminate	Aug 2019/ 11.89	Sept 2021/ Stage IA3 Adenocarcinoma	517
**3**	47%	Oct 2019/ 1.68 cm	Mar 2020/ Atypical-rare atypical cells	Mar 2020/ 2.85	May 2020/ N2 & N3 Small Cell Lung Cancer	60

Abbreviations: CT, computerized tomography scan

## Discussion

For individuals with IPNs, various clinical and radiologic factors have been found to be associated with higher risk of lung cancer [9]. The Mayo Clinic developed a highly validated and commonly used model that relies on an individual’s age, smoking status, history of cancer, and nodule characteristics to predict lung cancer risk in individuals with suspicious lung lesions [40, 41]. Assessment of these factors alone has proved insufficient for high confidence risk stratification of individuals with IPNs and there exists a need for additional independent predictors and biomarkers of lung cancer [9]. We applied the Mayo Clinic Model to characterize the participants in our study, as it is the most validated risk model [40, 41], and 15.9% of participants had high and 13.2% had low pre-test probability of lung cancer. The majority (70.9%) of participants fell into the intermediate-risk class, for which risk stratification in clinical practice remains challenging [9, 40]. Notably, the Mayo model performed poorly in our study. This is because the factors used in the Mayo model were indistinguishable between benign and malignant nodules in our study population, which is similar to the DECAMP study population described by Kammer et al. [42], where the AUC for the Mayo Model was 0.59. However, this population would derive the greatest benefit from improved lung cancer prediction tests evaluating novel biomarkers, such as the LungLB™ test, that can better inform clinical decision-making.

To evaluate the performance of the LungLB™ test, multivariate analysis revealed that CGACs were the strongest independent predictor of malignant lung cancer and improved predictive power was achieved when used in adjunct with clinical factors. This finding suggests that CGACs may serve as a useful biomarker, and integrating CGAC count with clinical factors in lung cancer risk assessments may provide superior results with greater confidence, although more work needs to be done to verify this [9]. In addition to risk models, guidelines recommend the use of fluorodeoxyglucose PET for indeterminate nodule evaluation in certain situations. PET utility has been called into question, especially for nodules < 2 cm and in areas of endemic granulomatous disease [43]. In our study, 52% of study participants had PET scan performed, and based on the commonly accepted cut-off of SUV_max_=2.5 [44], PET performed with 67.2% sensitivity and 44.4% specificity (AUC = 0.56). Further investigation and additional studies showing the clinical utility of LungLB™ would strengthen our understanding of its value.

The diagnostic value of Advanced CGACs was found to be greater than other CGACs in detecting malignant lung cancer. We discovered that Advanced CGACs displayed a larger average nuclear area compared with normal WBCs and other CGACs. Abnormal nuclear morphologies, including an enlarged nucleus, have been previously reported in malignant cells, which support our finding that Advanced CGACs are more highly correlated with malignancy in the LungLB™ test [45–47].

This discovery has additional implications for other diagnostic tests that detect CTCs. CTC enrichment commonly relies on isolation based on cell surface markers and size exclusion [48–51]. Therefore, CGACs without expression of traditional CTC surface markers or that fall outside of the isolation size window would be excluded in these tests. The LungLB™ test utilizes FISH to identify CGACs, which allows for more unbiased detection of CGACs regardless of their surface marker expression profile or size.

Our analyses revealed that the LungLB™ test displayed consistent and robust performance across various participant characteristics. The LungLB™ test continued to display robust performance even in participants with smaller (< 2 cm) nodules, sub/nonsolid nodules, and stage I disease, although the small number of participants with stage II-IV disease in this study warrants further investigation. Our finding that the LungLB™ test performed well in participants with stage I disease was surprising, given that other cancer detection tests typically exhibit poor performance with detecting early-stage disease [22, 30]. The LungLB™ test demonstrated 79.0% sensitivity in stage I lung cancer and 61.3% sensitivity across stage II-IV lung cancer. This finding is particularly impactful given that early-stage lung cancer is where there currently exists the greatest unmet need in terms of an accurate, minimally invasive diagnostic test. We propose that the higher performance associated particularly with subsolid, smaller-sized, earlier-stage malignant lesions may be related to their disease pathogenesis. Lung cancer has been shown to exhibit early metastatic behavior both clinically and in pre-clinical models of cells representative of premalignant lesions [17], which often manifest as subsolid lesions by CT. With tumor progression, fast growing cells in a tumor may outpace highly motile, slow-growing cells as the dominant cell type as described due to a “Grow or Go” phenotypic switch [52, 53], which may result in altered tumor-associated cells, such as CGAC, in circulation.

The majority of participants with malignant lesions had adenocarcinoma (> 65%); the low number of participants with squamous cell carcinoma and small cell/neuroendocrine carcinomas make it challenging to accurately assess test performance by lung cancer subtype. Future studies with expanded patient numbers are underway to further refine our understanding of performance in these subtypes.

The LungLB™ test also may offer earlier detection of lung cancer compared with current standard of care as well as sparing individuals from unnecessary biopsies (Table 3, stage I detection with 79.0% sensitivity and 74.4% specificity). In the described cases, initial biopsies indicated a negative or indeterminate diagnosis. Meanwhile, the LungLB™ test, which was performed concurrently with the biopsies, positively indicated lung cancer in each case. Later analysis of surgically resected tissue revealed that each patient had lung cancer. Notably in Case 2, surgical resection of the nodule did not occur until 2 years after the initial biopsy, which may have been due to COVID-related disruptions in care. Had LungLB™ been used in the nodule evaluation process, each of these patients may have received treatment months to years earlier than with the current diagnostic pathway. Although the volume doubling times of IPNs are highly variable, malignant lung cancer nodules are associated with rapid doubling times and aggressive metastasis; therefore, delays in proper diagnosis and treatment of malignant lung cancer have significant implications and may lead to poorer patient outcomes [54]. The inability to provide accurate diagnoses with initial biopsies also subjects patients to the burden of frequent follow-up screenings and examinations and continued anxiety, especially if the biopsy procedure resulted in an adverse event. This highlights the broad potential clinical utility of the LungLB™ test.

This study is not without limitations. All participant samples were collected from 2 sites and comprise a relatively limited sample set, which does not span the full diversity of patient demographics nor IPNs that exist. The follow-up range was broad (9–24 months), and while most participants had received a diagnosis within months, there were a minority of cases where diagnostic delays were COVID-related. Our multivariate analysis was performed to identify independent predictors of malignancy in this dataset; we do not propose it be used as a clinical model for nodule evaluation as this would require a separate validation. These models were developed using a small dataset and therefore there is risk of overfitting. Of importance to note is that one of the sites we enrolled participants was MD Anderson Cancer Center in Houston, Texas, which is in a region with higher incidence of fungal histoplasmosis infections. For clinicians, distinguishing infectious etiology from lung cancer is often challenging and presence of histoplasmosis infection can confound lung cancer diagnosis [43]. In our study, 11 (28.2%) of 39 participants with benign lesions had infectious etiology. The LungLB™ test maintained strong performance in these individuals with confounding lung infections, further demonstrating its promise in diagnosing lung cancer in challenging populations.

The LungLB™ test utilizes FISH, a highly specific and sensitive assay, to detect DNA CNVs that are a hallmark of cancer to broadly detect CGACs. However, because the test does not rely on the traditional markers of CTCs to identify CGACs, further characterization of CGACs is warranted and immunophenotyping of these cell populations is underway. Further characterization of these cells may help in understanding the pathogenesis of lung cancer, as well as provide the potential for identification of additional cell markers that may be used to further improve the performance of the LungLB™ test. Additionally, novel biomarker targets may also be revealed, which may be leveraged in the development of targeted therapies.

Our data indicate that the LungLB™ test, a minimally invasive liquid biopsy, FISH-based assay, may discriminate benign from malignant processes in individuals with IPNs at risk for lung cancer. We report that the LungLB™ test performs with high specificity and sensitivity, which may be further enhanced when combined with clinical factors. Additional studies of the LungLB™ test are warranted.

## Electronic supplementary material

Below is the link to the electronic supplementary material.

Supplementary Material 1

Supplementary Material 2

Supplementary Material 3

Supplementary Material 4

Supplementary Material 5

Supplementary Material 6

## Data Availability

The datasets generated during the current study are not publicly available due to concerns regarding participant confidentiality and proprietary information but are available upon reasonable request from the corresponding author.

## Abbreviations

CGACs: Circulating Genetically Abnormal Cells
CT: Computed Tomography
IPNs: Indeterminate Pulmonary Nodules
CTC: Circulating Tumor Cells
COPD: Chronic Obstructive Pulmonary Disease
ctDNA: Circulating Tumor DNA
cfDNA: Cell Free DNA
NSCLC: Non-Small Cell Lung Cancer
CNV: Copy Number Variation
FISH: Fluorescence *in situ* Hybridization
AUC: Area Under the Curve
ROC: Receiver Operating Characteristic
WBC: White Blood Cells
PET: Positron Emission Tomography
