# Supplementary material for: The presence of circulating genetically abnormal cells in blood predicts risk of lung cancer in individuals with indeterminate pulmonary nodules
Source: BMC Pulm Med. 2023 Jun 5;23:193. doi: 10.1186/s12890-023-02433-4 (PMC10240808; doi:10.1186/s12890-023-02433-4)
Supplement: Supplementary file 1 — Supplementary Material 1 [file 12890_2023_2433_MOESM1_ESM.docx]

**Figure S1. Study Participant Enrollment Flowchart**

**Enrollment (December 2018-February 2021)**

182 participants assessed for eligibility and enrolled

19 participants excluded

- 14 indeterminate lung
  nodule diagnoses
- 5 samples not processed
  (clotted or damaged)

**Initial Evaluation**

163 participants met evaluation criteria

12 participants excluded

- 12 samples did not pass assay QC criteria

**Final Analysis**

151 participants met all study inclusion criteria

- 112 (74.2%) with confirmed malignant nodule
- 39 (25.8%) with confirmed benign nodule

Between December 2018 and February 2021, 182 participants were assessed for study eligibility and enrolled. Nineteen participants were excluded; 14 were excluded due to indeterminate lung nodule diagnosis and 5 were excluded due to the inability to process the respective participant’s blood sample. Of the remaining 163 participants, 12 additional participants were excluded because their respective blood sample did not pass the assay’s quality control (QC) criteria. A total of 151 participants were included in the study’s final analysis and 112 participants had confirmed malignant nodules and 39 participants had confirmed benign nodules.
