# Supplementary material for: The presence of circulating genetically abnormal cells in blood predicts risk of lung cancer in individuals with indeterminate pulmonary nodules
Source: BMC Pulm Med. 2023 Jun 5;23:193. doi: 10.1186/s12890-023-02433-4 (PMC10240808; doi:10.1186/s12890-023-02433-4)
Supplement: Supplementary file 2 — Supplementary Material 2 [file 12890_2023_2433_MOESM2_ESM.docx]

**Figure S2. Normalized Nuclear Area of CGACs and Advanced CGACs from Benign and Malignant Participant Samples**

**
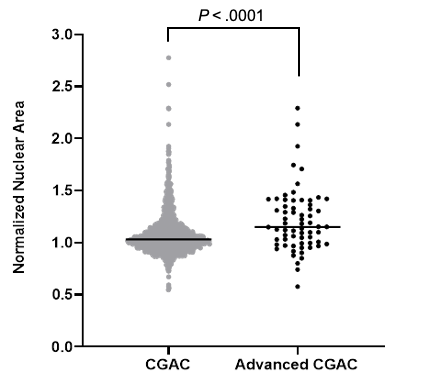
**

Abbreviations: CGAC, circulating genetically abnormal cell; WBC, white blood cell.

The normalized nuclear area for CGACs (gray dots, n = 1528) and Advanced CGACs (black dots, n = 65) is shown. The nuclear areas of CGACs and Advanced CGACs were normalized to the average nuclear area of normal WBCs from each respective participant’s sample. Normalized nuclear area values >1 indicate a larger nuclear area compared with normal WBCs; normalized nuclear area values <1 indicate smaller nuclear area compared with normal WBCs. The horizontal black bar represents the median normalized nuclear area, which was 1.03 for CGACs and 1.15 for Advanced CGACs (mean normalized nuclear area was 1.07 for CGACs vs 1.21 for Advanced CGACs).
