# Supplementary material for: The presence of circulating genetically abnormal cells in blood predicts risk of lung cancer in individuals with indeterminate pulmonary nodules
Source: BMC Pulm Med. 2023 Jun 5;23:193. doi: 10.1186/s12890-023-02433-4 (PMC10240808; doi:10.1186/s12890-023-02433-4)
Supplement: Supplementary file 3 — Supplementary Material 3 [file 12890_2023_2433_MOESM3_ESM.docx]

**Figure S3. Calibration Curves for LungLB Unweighted and Weighted**


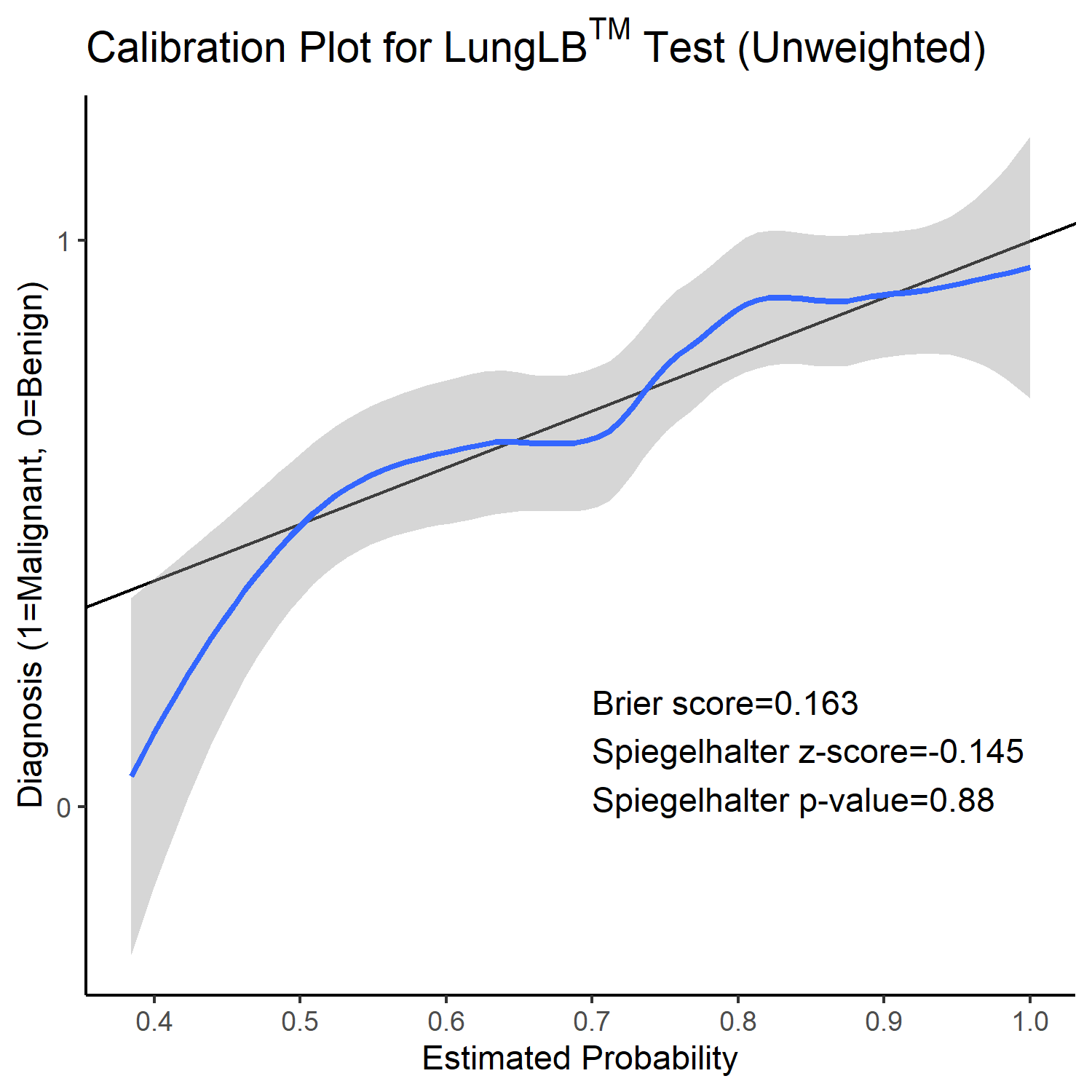


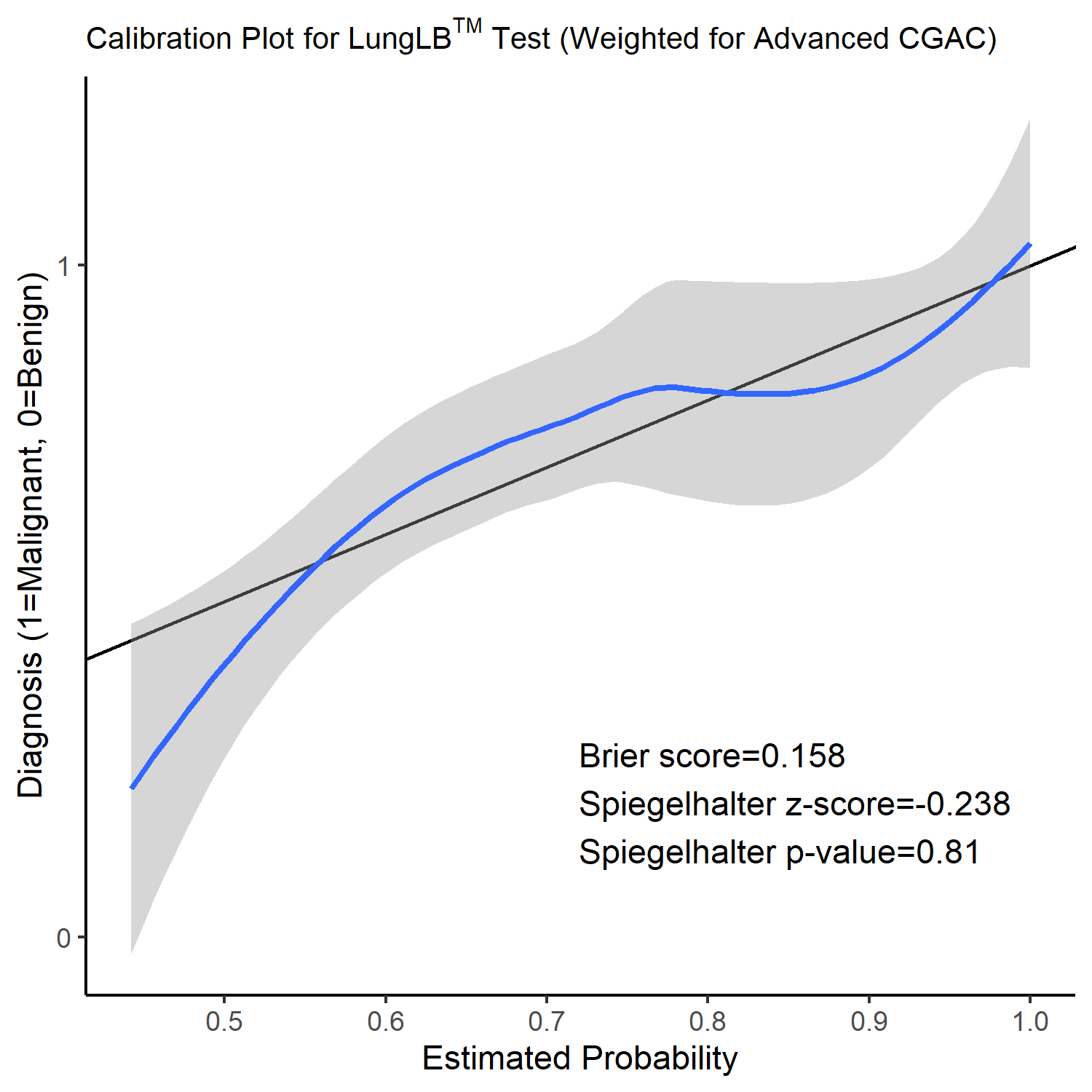


Calibration curves were created by plotting predicted probabilities from the logistic regression model against the dichotomized diagnosis outcome and fitting the curve using the loess smoother. Brier score, Spiegelhalter scores and p-values were calculated using the ‘rms’ package in R. Decision curves were created using the ‘dcurves’ package in R.

For each calibration plot, the diagonal line indicates perfect calibration, the blue line indicates the calibration of the LungLB test (unweighted and weighted for advanced CGACs). The Brier score is the mean squared error between the predicted probabilities and the actual outcomes, this score ranges from 0 to 1, with a score of 0 being a perfect fit and a score of 1 representing an imperfect fit. A Brier score of 0.163 (unweighted) and 0.158 (weighted) indicates that the LungLB assay is a good fit. The Spiegelhalter z-score is derived from the Brier score and is used to calculate the Spiegelhalter p-value. The p-value tests for misclassification, therefore a p-value <0.05 would indicate that our model is poorly calibrated. The LungLB assay has a p-value of 0.88 (unweighted) and 0.81 (weighted) indicating that the LungLB assay is calibrated properly.
