# Supplementary material for: The presence of circulating genetically abnormal cells in blood predicts risk of lung cancer in individuals with indeterminate pulmonary nodules
Source: BMC Pulm Med. 2023 Jun 5;23:193. doi: 10.1186/s12890-023-02433-4 (PMC10240808; doi:10.1186/s12890-023-02433-4)
Supplement: Supplementary file 4 — Supplementary Material 4 [file 12890_2023_2433_MOESM4_ESM.docx]

**Table S1. Normalized Nuclear Area of CGACs and Advanced CGACs From All Participant Samples**

| **Category** | **n** | **Median (IQR)** | **Mean (95% CI)** | **SD** | **SEM** | ***P* value** |
| --- | --- | --- | --- | --- | --- | --- |
| CGAC | 1528 | 1.032  (0.547-1.130) | 1.072  (1.063-1.080) | 0.185 | 0.004 | <.0001 |
| Advanced CGAC | 65 | 1.152  (0.995-1.385) | 1.211  (1.136-1.286) | 0.302 | 0.038 |  |

Abbreviations: CGAC, circulating genetically abnormal cell; CI, confidence interval; IQR, interquartile range; SD, standard deviation; SEM, standard error of the mean; WBC, white blood cell.

The nuclear areas of CGACs and Advanced CGACs were normalized to the average nuclear area of normal WBCs from each respective participant sample. Median (IQR) and mean (95% CI, SD, SEM) normalized nuclear area of CGACs and Advanced CGACs are presented. Normalized nuclear area values >1 indicate a larger nuclear area compared with normal WBCs; normalized nuclear area values <1 indicate smaller nuclear area compared with normal WBCs. The normalized nuclear area of Advanced CGACs was significantly larger than CGACs (*P* < .0001).
