# Supplementary material for: The presence of circulating genetically abnormal cells in blood predicts risk of lung cancer in individuals with indeterminate pulmonary nodules
Source: BMC Pulm Med. 2023 Jun 5;23:193. doi: 10.1186/s12890-023-02433-4 (PMC10240808; doi:10.1186/s12890-023-02433-4)
Supplement: Supplementary file 5 — Supplementary Material 5 [file 12890_2023_2433_MOESM5_ESM.docx]

**Table S2. Multivariate Analyses to Identify Independent Predictors of Malignancy in the LungLB™ Test**

| **Model 1** | **AUC** | **OR (95% CI)** | ***P*-value** |
| --- | --- | --- | --- |
| **CGAC (Unweighted)** | **0.74** | 1.81 (1.35-2.45) | <.001 |
|  |  |  |  |
| **Model 2** | **AUC** | **OR (95% CI)** | ***P*-value** |
| **CGAC (Weighted for Advanced CGACs)** | **0.78** | 1.44 (1.20-1.72) | <.001 |
|  |  |  |  |
| **Model 3** | **AUC** | **OR (95% CI)** | ***P*-value** |
| **Clinical Factors** | **0.67** |  |  |
| Age |  | 1.00 (0.94-1.06) | .97 |
| Male |  | 1.45 (0.54-3.89) | .46 |
| Ever Smoker |  | 0.77 (0.18-3.20) | .72 |
| COPD/Emphysema |  | 0.45 (0.16-1.26) | .13 |
| Subsolid Nodules |  | 3.08 (0.91-10.43) | .07 |
| Upper Nodule Location |  | 0.80 (0.29-2.19) | .66 |
| Nodule Size |  | 1.40 (0.85-2.33) | .19 |
| Cancer History |  | 1.01 (0.34-3.00) | .99 |
|  |  |  |  |
| **Model 4** | **AUC** | **OR (95% CI)** | ***P*-value** |
| **CGAC (Unweighted) + Clinical Factors** | **0.74** |  |  |
| CGAC (Unweighted) |  | 1.65 (1.14-2.38) | .01 |
| Age |  | 0.98 (0.92-1.04) | .53 |
| Male |  | 1.57 (0.56-4.41) | .39 |
| Ever Smoker |  | 0.92 (0.20-4.16) | .91 |
| COPD/Emphysema |  | 0.52 (0.18-1.53) | .24 |
| Subsolid Nodules |  | 3.20 (0.85-12.02) | .09 |
| Upper Nodule Location |  | 0.66 (0.22-1.97) | .46 |
| Nodule Size |  | 1.56 (0.91-2.65) | .10 |
| Cancer History |  | 0.98 (0.31-3.16) | .98 |
|  |  |  |  |
| **Model 5** | **AUC** | **OR (95% CI)** | ***P*-value** |
| **CGAC (Weighted for Advanced CGACs) + Clinical Factors** | **0.78** |  |  |
| CGAC (Weighted for Advanced CGAC) |  | 1.36 (1.11-1.66) | .00 |
| Age |  | 0.99 (0.93-1.06) | .84 |
| Male |  | 1.57 (0.54-4.51) | .41 |
| Ever Smoker |  | 0.53 (0.11-2.47) | .41 |
| COPD/Emphysema |  | 0.60 (0.20-1.83) | .37 |
| Subsolid Nodule |  | 3.44 (0.93-12.66) | .06 |
| Upper Nodule Location |  | 0.88 (0.30-2.61) | .82 |
| Nodule Size |  | 1.64 (0.95-2.84) | .08 |
| Cancer History |  | 1.09 (0.34-3.50) | .89 |
|  |  |  |  |
| **Model 6** | **AUC** | **OR (95% CI)** | ***P*-value** |
| **CGAC (Unweighted) + Nodule Size** | **0.76** |  |  |
| CGAC (Unweighted) |  | 1.78 (1.31-2.43) | <.001 |
| Nodule Size |  | 1.47 (1.00-2.18) | .05 |
|  |  |  |  |
| **Model 7** | **AUC** | **OR (95% CI)** | ***P*-value** |
| **CGAC (Weighted for Advanced CGACs) + Nodule Size** | **0.78** |  |  |
| CGAC (Weighted for Advanced CGACs) |  | 1.42 (1.19-1.71) | <.001 |
| Nodule Size |  | 1.48 (0.99-2.21) | .05 |

Abbreviations: AUC, area under the curve; CI, confidence interval; COPD, chronic obstructive pulmonary disease; OR, odds ratio.

Multivariate analyses were performed to determine independent predictors of lung cancer. Seven different models were established to identify independent predictors of lung cancer and to determine whether different combination of these variables may improve overall LungLB™ test performance. The independent predictors that were evaluated included CGAC (Model 1), CGAC with weighting of Advanced CGACs (Model 2), Clinical Factors (Model 3), CGAC + Clinical Factors (Model 4), CGAC with weighting of Advanced CGACs + Clinical Factors (Model 5), CGAC + Nodule Size (Model 6), and CGAC with weighting of Advanced CGACs + Nodules Size (Model 7).
