# Supplementary material for: The presence of circulating genetically abnormal cells in blood predicts risk of lung cancer in individuals with indeterminate pulmonary nodules
Source: BMC Pulm Med. 2023 Jun 5;23:193. doi: 10.1186/s12890-023-02433-4 (PMC10240808; doi:10.1186/s12890-023-02433-4)
Supplement: Supplementary file 6 — Supplementary Material 6 [file 12890_2023_2433_MOESM6_ESM.docx]

**Table S3. DeLong Comparison of the Multivariate Models Evaluating Independent Predictors of Malignancy in the LungLB™ Test**

| **Models Comparison** | ***P*-value** |
| --- | --- |
| **1 vs 2** | .208 |
| **1 vs 3** | .270 |
| **1 vs 4** | .949 |
| **1 vs 5** | .672 |
| **1 vs 6** | .911 |
| **1 vs 7** | .598 |
| **2 vs 3** | .104 |
| **2 vs 4** | .639 |
| **2 vs 5** | .903 |
| **2 vs 6** | .657 |
| **2 vs 7** | .977 |
| **3 vs 4** | .106 |
| **3 vs 5** | **.053** |
| **3 vs 6** | .233 |
| **3 vs 7** | .115 |
| **4 vs 5** | .567 |
| **4 vs 6** | .966 |
| **4 vs 7** | .664 |
| **5 vs 6** | .758 |
| **5 vs 7** | .927 |
| **6 vs 7** | .401 |

Abbreviations: AUC, area under the curve.

The DeLong test was used to compare the AUCs between various multivariate models presented in Table S2. No statistically significant differences were identified between multivariate models, but comparison of Models 3 vs 5 demonstrated a *P* = .053.
